# Genome-wide association study to identify genomic regions and positional candidate genes associated with male fertility in beef cattle

**DOI:** 10.1038/s41598-020-75758-3

**Published:** 2020-11-18

**Authors:** H. Sweett, P. A. S. Fonseca, A. Suárez-Vega, A. Livernois, F. Miglior, A. Cánovas

**Affiliations:** 1 Department of Animal Biosciences, Centre for Genetic Improvement of Livestock, University of Guelph, Guelph, ON N1G 2W1 Canada; 2 Department of Pathobiology, Ontario Veterinary College, University of Guelph, Guelph, ON N1G 2W1 Canada

**Keywords:** Agricultural genetics, Animal breeding, Functional genomics, Genetic association study

## Abstract

Fertility plays a key role in the success of calf production, but there is evidence that reproductive efficiency in beef cattle has decreased during the past half-century worldwide. Therefore, identifying animals with superior fertility could significantly impact cow-calf production efficiency. The objective of this research was to identify candidate regions affecting bull fertility in beef cattle and positional candidate genes annotated within these regions. A GWAS using a weighted single-step genomic BLUP approach was performed on 265 crossbred beef bulls to identify markers associated with scrotal circumference (SC) and sperm motility (SM). Eight windows containing 32 positional candidate genes and five windows containing 28 positional candidate genes explained more than 1% of the genetic variance for SC and SM, respectively. These windows were selected to perform gene annotation, QTL enrichment, and functional analyses. Functional candidate gene prioritization analysis revealed 14 prioritized candidate genes for SC of which *MAP3K1* and *VIP* were previously found to play roles in male fertility. A different set of 14 prioritized genes were identified for SM and five were previously identified as regulators of male fertility (*SOD2, TCP1, PACRG, SPEF2, PRLR*). Significant enrichment results were identified for fertility and body conformation QTLs within the candidate windows. Gene ontology enrichment analysis including biological processes, molecular functions, and cellular components revealed significant GO terms associated with male fertility. The identification of these regions contributes to a better understanding of fertility associated traits and facilitates the discovery of positional candidate genes for future investigation of causal mutations and their implications.

## Introduction

As the human population continues to grow exponentially, so does the demand for animal proteins, such as beef and beef by-products^1^. In 2014, the Intergovernmental Panel on Climate Change (IPCC) report estimated that by 2050 meat consumption will rise 21% per capita as a result of a larger human population, leading to business and economic benefits for beef producers^2^. One way we can continue to match the demand of beef and beef by-products with the ever-growing population is through increasing beef cattle fertility^3,4^. This can be attained through the use of genetics to select for more fertile bulls, thereby increasing breeding success rates and decreasing the number of replacement heifers. However, over the last few decades, fertility in beef cattle has decreased because of intense selection pressure on production traits^3^. One of the reasons for this decrease in fertility results from the pleiotropic effect of genes and biological processes underlying fertility and production, such as cell proliferation and energy conservation metabolism^5,6^. Therefore, further research on the functional biology underlying fertility traits in cattle is required to prevent these undesirable side effects of selection^6–9^.

Genetic studies have focused primarily on female fertility, which presents a significant challenge to improving beef cattle fertility because of the low heritability (0.01–0.10) of these reproductive traits^10^. Fertility-related traits in bulls are moderately heritable (0.05–0.22) and significantly influenced by genetics^11^. Therefore, improving our understanding of the genes and epigenetic modifications that contribute to bull fertility could improve reproduction success in beef cattle^11,12^. Indeed, sires directly influence the fertilization process, the viability of the preimplantation embryo, as well as the conception rate^13^. Sperm motility (SM) could be used as a proxy for identifying more fertile sires and improving reproduction success because it is a moderate to highly heritable trait (0.29–0.60) with significant components in the genome contributing to semen quality^14–16^. With reduced motility in bull semen becoming a prominent concern in the breeding industry, it is essential to continue studying genomic regions, variants and functional genes that impact motility^15^.

Scrotal circumference (SC) is also a good indicator trait for sire fertility because it is highly correlated with testes weight, sperm output, and semen quality^17^. Previous studies have suggested that a smaller SC is associated with lower fertility measures compared to bulls with a larger SC^18^. Moreover, a positive association has been reported between SC and the percentage of live sperm, sperm number, and SM^18^. Therefore, the identification of genetic markers linked to SC and SM could be used as a strategy to genetically improve reproductive performance^17^. However, there is a negative genetic correlation between performance traits, such as feed efficiency, and SC, with more feed-efficient bulls having smaller SC^17,19^. Thus, careful consideration must be taken into account when selecting for both production and fertility traits to avoid undesirable effects.

Relatively fewer GWAS for bull fertility traits have focused on crossbred beef cattle, which represent a significant proportion of the beef cattle population, compared to purebred cattle^20,21^. This is in part due to multiple beef breeds with each breed association carrying out a separate genetic evaluation using different methods to calculate expected progeny deviation and the correspondence of SNP effects^22^. In this study we used a sample size of 265 bulls with both SC and SM measurements. Numerous other male fertility GWAS in cattle have obtained interesting results with a very similar sample size^15,18,23–27^. Therefore, the objectives of the present study were (1) to find SNP windows significantly associated with SC and SM in crossbred beef bulls using a single-step approach, and (2) to identify positional candidate genes with additional functional evidence and their potential role in bull fertility. To the best of our knowledge, this is the first study that uses a GWAS to analyze both SC and SM measurements simultaneously, in Canadian crossbred beef cattle, bringing new considerations for the current stage of literature regarding male fertility traits in beef cattle.

## Results

### Scrotal circumference genome wide association study and QTL enrichment

**Figure 1:**
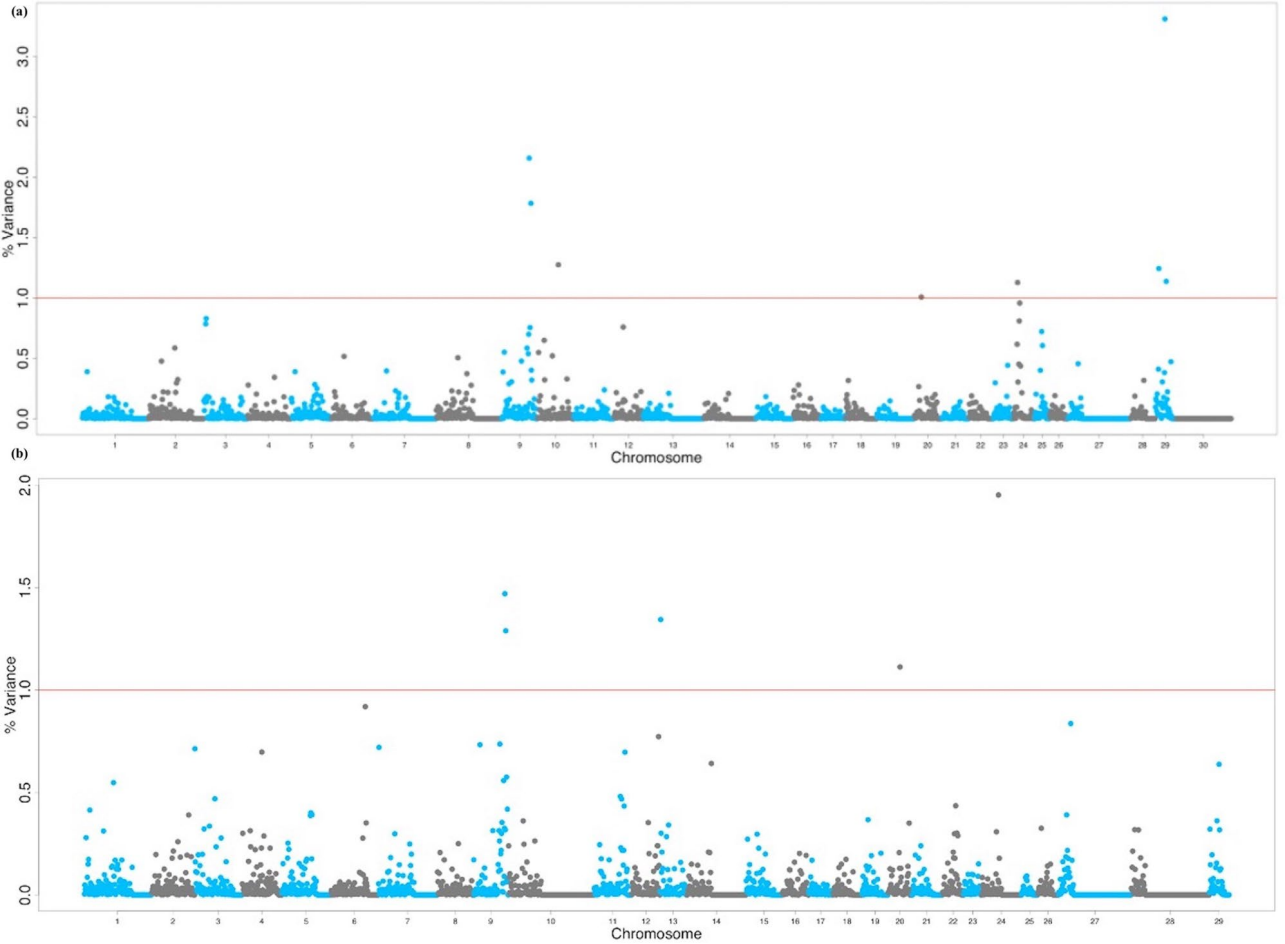
Manhattan plot for the GWAS of (**a**) scrotal circumference and (**b**) sperm motility in crossbred beef cattle. The y-axis shows the proportion of variance explained by the non-overlapping windows and the x-axis indicates the chromosome number. The red line indicates the threshold for 1% of the variance explained by the windows.

**Figure 2:**
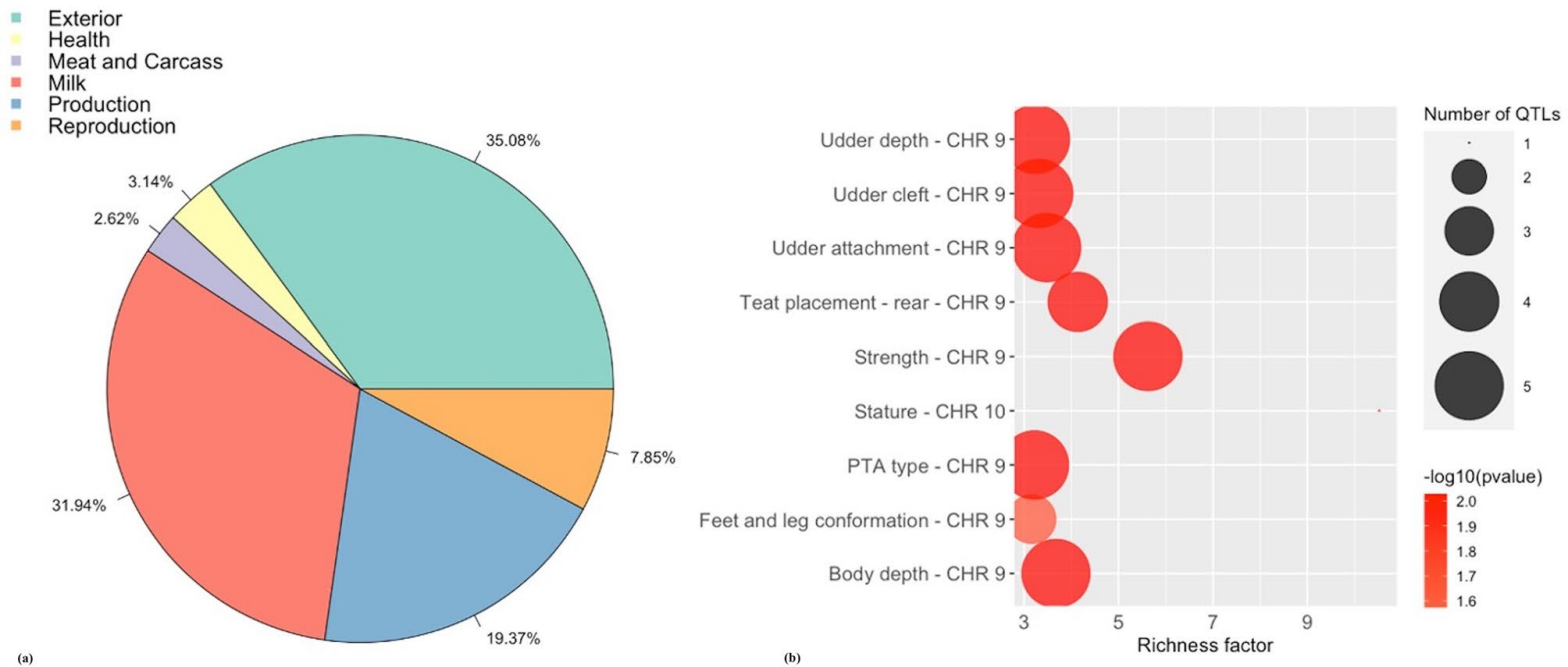
(**a**) Pie plot showing the percentage of each QTL class annotated in the windows explaining > 1% of the total genetic variance for scrotal circumference. (**b**) Enriched traits identified in the QTL enrichment analysis for scrotal circumference. The area with the circles represents the number of observed QTLs for that class, while the color represents the *p *value scale (the darker the color, smaller the *p *value). The x-axis shows the richness factor for each QTL, representing the ratio of number of QTLs and the expected number of that QTL.

After quality control, 379,591 markers remained for analysis based on a call rate greater than 95% and a minor allele frequency greater than 5% with non-autosomal markers. The Manhattan plot in Fig. 1a shows the non-overlapping windows 1 Mb apart which explain the highest proportion of variance for SC. Eight windows explained more than 1% of the genetic variance for SC which were located on BTA9, BTA10, BTA20, BTA24 and BTA29, and explained 13.19% of the total genetic variance (Table 1). Thirty-two positional candidate genes were identified within these windows explaining more than 1% of the genetic variance for SC. ToppGene prioritization analysis revealed 14 of the 32 positional candidate genes were prioritized for spermatic-related processes and the windows which these genes are mapped within explained 9.76% of the total genetic variance for SC. The QTL annotation revealed several reproduction QTLs, annotated within the coordinates of the windows explaining > 1% of the total genetic variance for SC, account for 7.85% of the QTLs annotated in those regions (Fig. 2a). The QTL enrichment analysis revealed 38 significant QTLs (FDR-corrected *p* value ≤ 0.05) on BTA9 and BTA10 annotated for traits related to exterior conformation (Table 2; Fig. 2b).

**Table 1:** Significant associated windows explaining more than 1% of the variance for scrotal circumference and sperm motility in crossbreed beef cattle and the overlapping genes located within those windows.

Trait	Window region			% Variance	Gene names
	BTA	Start	End		
Scrotal circumference	9	85,069,592	86,068,330	2.42	SASH1*, UST
	9	89,514,561	90,513,278	1.61	RF00100, MYCT1, VIP*, FBXO5*, MTRF1L*, RGS17*, bta-mir-2480
	10	62,210,731	63,210,223	1.26	MYEF2, SLC24A5*, SEMA6D*
	20	22,183,762	23,180,410	1.00	MIER3, SETD9, RF00003, MAP3K1*, *RF00026, ANKRD55*
	24	10,933,271	11,930,436	1.04	*RF00001*
	29	10,283,051	11,269,974	1.20	*RF02113, RF02112, bta-mir-2285 k-1*
	29	29,347,332	30,347,114	3.47	RPUSD4, FAM118B, SRPRA*, FOXRED1, TIRAP*, DCPS*, ST3GAL4*, KIRREL3*, CDON*
	29	33,525,642	34,523,872	1.18	*OPCML*
Sperm motility	9	95,179,163	96,178,394	1.47	bta-mir-12055, RSPH3, TAGAP, FNDC1, SOD2*, WTAP, ACAT2*, TCP1*, RF00401, RF00429, MRPL18, PNLDC1, MAS1*, EZR*
	9	97,251,687	98,242,399	1.29	bta-mir-2482, PRKN*, PACRG*
	13	1,818,757	2,813,363	1.34	PLCB4*, LAMP5, PAK5*
	20	38,077,292	39,076,620	1.11	LMBRD2, UGT3A2, CAPSL, IL7R*, SPEF2*, SKP2*, PRLR*
	24	51,881,818	52,880,825	1.95	DCC*

*Prioritized candidate gene identified by GUILDify and ToppGene analysis.

**Table 2:** Significantly enriched QTLs identified annotated in the windows explaining more than 1% of the variance for scrotal circumference and sperm motility.

Trait	QTL Type	BTA	Number of QTLs	Expected number of QTLs	*p *value	FDR-corrected *p *value
Scrotal circumference	Udder depth	9	5	1.54	3.00 × 10^–3^	9.38 × 10^–3^
	Body depth	9	5	1.36	5.00 × 10^–3^	9.38 × 10^–3^
	Feet and leg conformation	9	3	0.95	1.60 × 10^–2^	2.67 × 10^–2^
	PTA type	9	5	1.55	2.00 × 10^–3^	9.38 × 10^–3^
	Udder attachment	9	5	1.44	4.00 × 10^–3^	9.38 × 10^–3^
	Strength	9	5	0.89	1.00 × 10^–3^	9.38 × 10^–3^
	Teat placement—rear	9	4	0.97	3.00 × 10^–3^	9.38 × 10^–3^
	Udder cleft	9	5	1.51	4.00 × 10^–3^	9.38 × 10^–3^
	Stature	10	1	0.10	5.00 × 10^–3^	9.38 × 10^–3^
Sperm motility	Body weight gain	9	3	0.38	1.00 × 10^–3^	9.00 × 10^–3^
	Daughter pregnancy rate	9	1	0.34	4.50 × 10^–2^	8.10E−02
	Interval to first estrus after calving	9	2	0.61	2.50 × 10^–2^	8.10 × 10^–2^
	Milk fat yield	13	4	2.08	4.30 × 10^–2^	8.10 × 10^–2^
	Stature	13	3	1.13	3.50 × 10^–2^	8.10 × 10^–2^

^a^False discovery rate.

### Sperm motility genome wide association study and QTL enrichment

**Figure 3:**
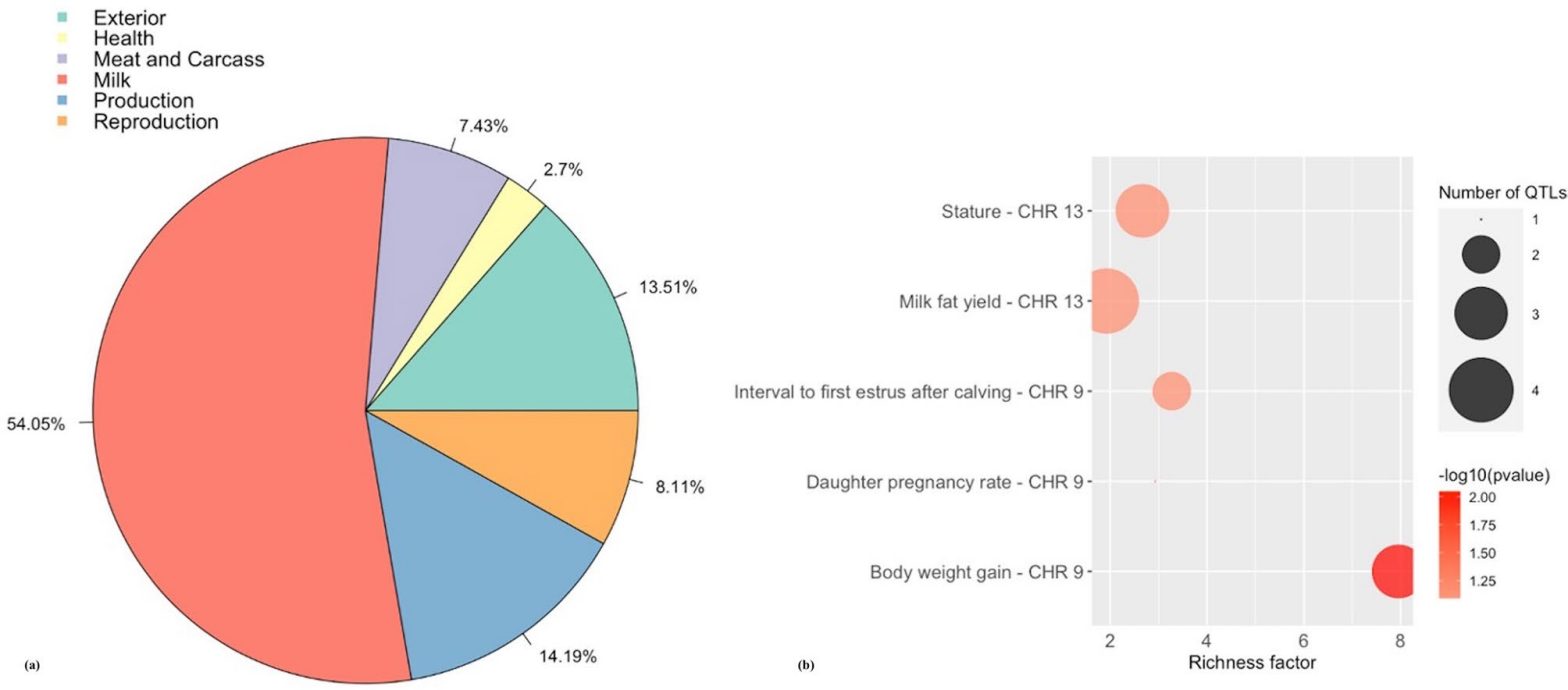
(**a**) Pie plot showing the percentage of each QTL class annotated in the windows explaining > 1% of the total genetic variance for sperm motility. (**b**) Enriched traits identified in the QTL enrichment analysis for sperm motility. The area with the circles represents the number of observed QTLs for that class, while the color represents the *p *value scale (the darker the color, smaller the *p *value). The x-axis shows the richness factor for each QTL, representing the ratio of number of QTLs and the expected number of that QTL.

The Manhattan plot in Fig. 1b shows the non-overlapping SNP windows 1 Mb apart that explain the highest proportion of variance for SM. Five windows explained more than 1% of the genetic variance for SM which were located on BTA9, BTA13, BTA20, and BTA24, and explained 7.17% of the total genetic variance (Table 1). Twenty-eight positional candidate genes were identified within these SNP windows explaining more than 1% of the genetic variance for SM. ToppGene prioritization revealed 14 of the 28 positional candidate genes were prioritized for spermatic-related processes and the windows which these genes are mapped within explained 7.16% of the total genetic variance for SM. The QTL annotation revealed several reproduction QTLs, annotated within the coordinates of the windows explaining > 1% of the total genetic variance for SM, account for 8.11% of the QTLs annotated in these regions (Fig. 3a). The QTL enrichment analysis revealed 13 significant QTLs (FDR-corrected *p* value ≤ 0.05) on BTA9 and BTA13 annotated for traits related to reproduction and exterior conformation (Table 2; Fig. 3b).

### Functional analysis of prioritized candidate genes for scrotal circumference and sperm motility

**Figure 4:**
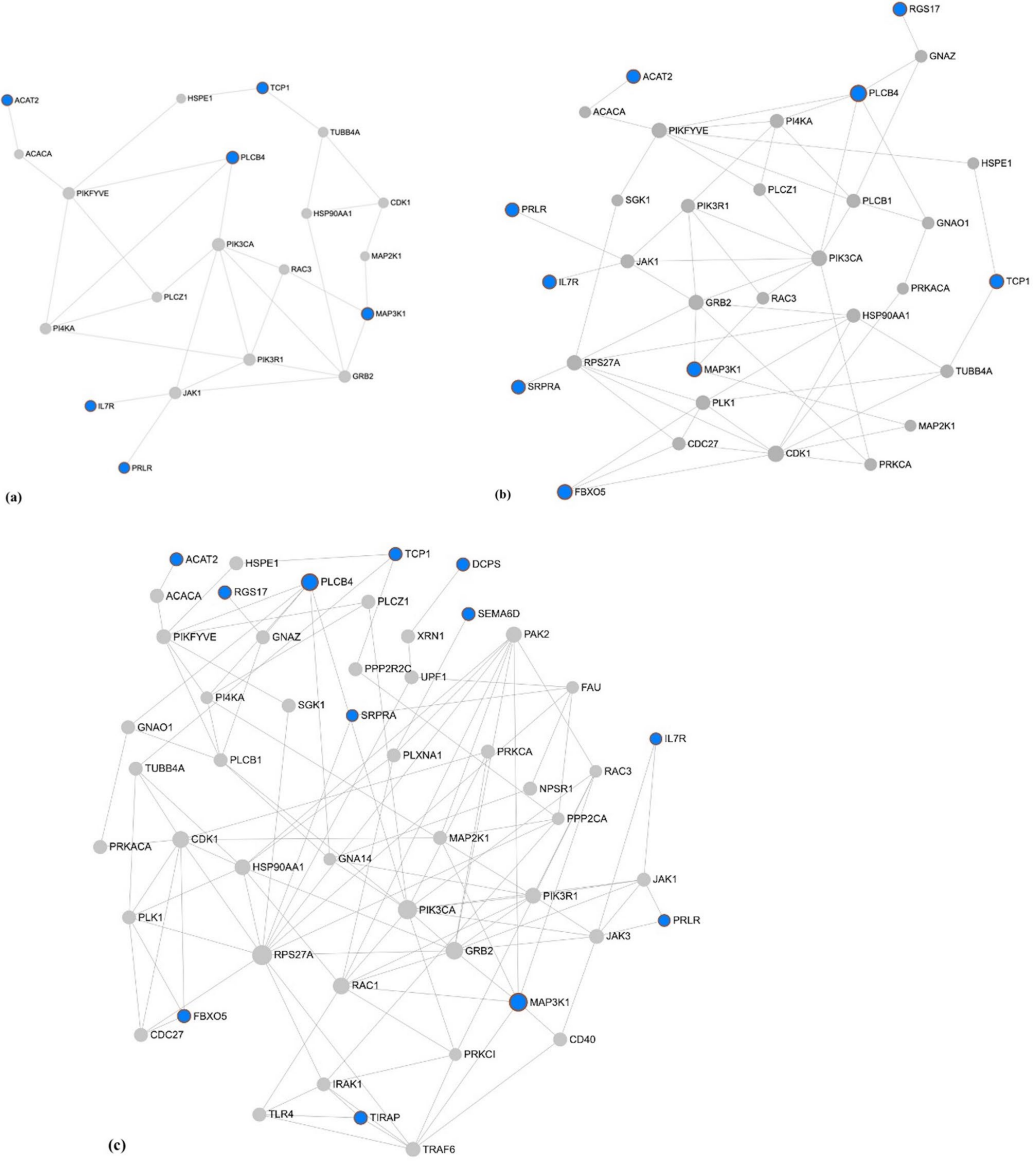
Gene network generated in NetworkAnalyst 3.0 (https://www.networkanalyst.ca) via selection of the prioritized candidate genes for scrotal circumference and sperm motility and the significant (**a**) Biological process, (**b**) Molecular function, and (**c**) Cellular component GO terms involved in reproductive processes (FDR-corrected *p *value ≤ 0.05).The blue circles are the prioritized candidate genes for scrotal circumference and sperm motility and the grey circles represent the directly connected genes.

The network constructed with the 14 prioritized candidate genes for SC and SM (total of 28 genes) using NetworkAnalyst had 1442 nodes (genes) related to 120 GO:BP (Table S2a), 78 GO:MF (Table S3a) and 69 GO:CC (Table S4a) terms. One hundred and twenty GO:BP were significant (FDR-corrected *p* value ≤ 0.05) and 64 were identified as related to male fertility and reproduction (Table S2b). These 64 GO:BP terms were selected to construct the first module containing 752 genes (Table S2c). A second module was generated composed only by the prioritized candidate genes for SC and SM and its directly connected nodes leading to a network constructed of 20 genes and 26 GO:BP terms (Fig. 4a, Table S2d). None of the GO:BP in this module were significant (the smallest FDR-corrected *p* value was 0.128), however nine were related to male fertility and reproduction of which regulation of *MAPK cascade*, *spermatid differentiation*, and *regulation of hormone secretion* were the most notable (Table 3). In this second module, five of the 20 genes were prioritized candidate genes for SM and one gene was prioritized for SC.

**Table 3:** List of significant gene ontology terms associated with biological processes, molecular functions, and cellular components related to fertility identified by NetworkAnalyst using the protein–protein interaction network built by the prioritized candidate genes for scrotal circumference and sperm motility and the directly connected nodes.

GO category	GO term	FDR^a^-corrected * p *value	Gene names
Biological process	Regulation of MAPK cascade	8.13 × 10^–1^	TCP1*, PIK3R1
	Spermatid differentiation	1	PRLR*
	Regulation of hormone secretion	1	*ACACA*
	Acetyltransferase activity	8.88 × 10^–4^	*CDC27, JAK1, GRB2, PIK3R1*
	Zinc ion binding	2.12 × 10^–3^	*JAK1, PIK3CA, PLK1, CDK1, PI4KA, SGK1, PRKACA*
Molecular function	Lipase activity	2.55 × 10^–3^	GRB2, MAP2K1, PLK1, MAP3K1*, PRKACA, FBXO5*
	Endonuclease activity	2.55 × 10^–3^	PIK3CA, PLK1, CDK1, MAP3K1*, SGK1, PRKACA
	Nuclease activity	4.58 × 10^–3^	JAK1, MAP2K1, PLK1, CDK1, MAP3K1*, SGK1, PRKACA
	Cation channel activity	9.51 × 10^–3^	PLCZ1, PLCB1, PLCB4*
	Kinesin complex	1.53 × 10^–2^	*CDC27, PLK1, CDK1*
Cellular component	Spindle microtubule	1.53 × 10^–2^	RAC1, PIK3CA, GRB2, TRAF6, GNA14, MAP2K1, IRAK1, TLR4, TIRAP*, PI4KA, HSP90AA1, XRN1, SGK1, PRKACA, PRLR*, CD40, NPSR1, PPP2CA, RGS17*
	Cytosol	4.42 × 10^–2^	*JAK1, JAK3, TUBB4A, PLK1, CDK1, PPP2CA*

^a^False discovery rate.

*Prioritized candidate gene identified by GUILDify and ToppGene analysis.

Of the 78 GO:MF, 75 were significant (FDR-corrected *p* value ≤ 0.05) and 34 were identified as related to male fertility and reproduction functions (Table S3b). The first module for GO:MF terms was extracted using these 34 GO:MF terms containing 684 genes (Table S3c). A second module for GO:MF was generated composed only by the prioritized candidate genes for SC and SM and its directly connected nodes revealing a network constructed of 32 genes and 20 GO:MF terms (Fig. 4b, Table S3d). In this second module, 10 GO:MF were significant (FDR-corrected *p* value ≤ 0.05) and six of these were related to male fertility and reproduction including, *acetyltransferase activity*, *zinc ion binding*, *lipase activity*, *endonuclease activity*, *nuclease activity*, and *cation channel activity* (Table 3). Five of the 32 genes in this second module for GO:MF were prioritized candidate genes for SM and four were prioritized candidate genes for SC.

**Figure 5:**
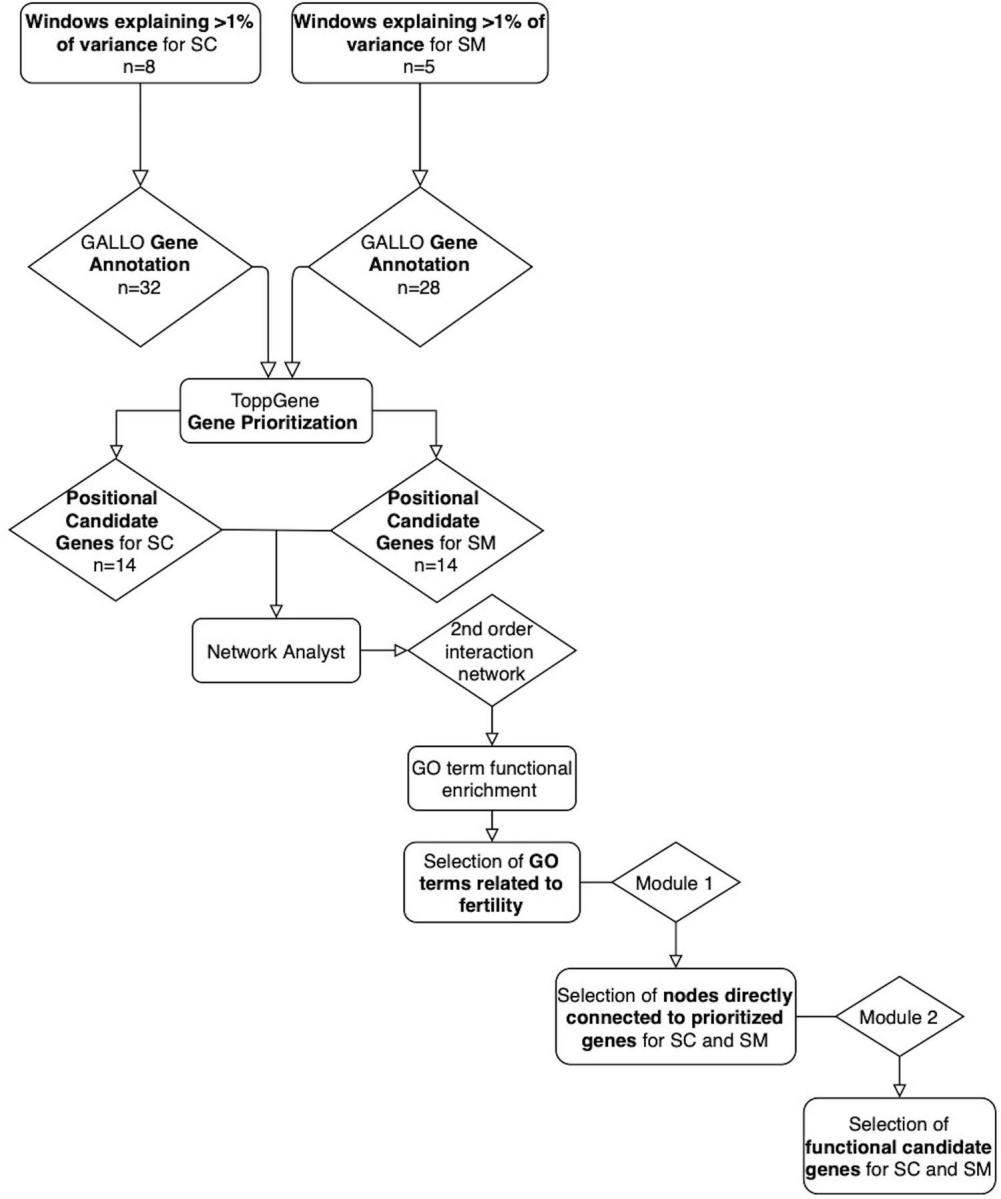
Graphical representation of the functional analyses performed to identify the positional candidate genes with additional functional evidence for scrotal circumference and sperm motility.

Of the 69 GO:CC terms, 55 were significant (FDR-corrected *p* value ≤ 0.05) and 28 were related to male fertility and reproduction functions and processes (Table S4b). These 28 GO:CC terms were selected to extract the first module for GO:CC which contained 992 genes (Table S4c). “Batch Selection” of the prioritized candidate genes for SC and SM was used to extract the second module for GO:CC which revealed a network containing 51 genes and 21 GO:CC terms (Fig. 4c, Table S4d). In this second module, five GO:CC were significant (FDR-corrected *p* value ≤ 0.05) and three of these were related to male fertility and reproduction including, *kinesin complex*, *spindle microtubule* and *cytosol* (Table 3). Five of the 51 genes in the second GO:CC module were prioritized candidate genes for SM and seven genes were prioritized candidate genes for SC. A graphical representation of the functional analysis can be found in Fig. 5.

## Discussion

The ability to identify cattle with greater reproductive performance would significantly improve the efficiency of the beef cattle industry. Thus, the discovery of genetic markers related to male fertility through GWAS can contribute to the evaluation of fertility traits, such as SC and SM, and their implications on male fertility. Spermatic related traits, including SM are commonly measured in dairy cattle where semen is widely used for artificial insemination and measuring semen parameters is a standard practice^15,21,24–27^. However, SM measurements are much less common in the beef industry where natural breeding is often used, which can be seen in our sample size. Testicular related traits, including SC have higher heritability values and correlations with fertility traits, although the majority of studies on SC have been on purebred bulls^18,21,28–32^. Few SC GWAS have been conducted in crossbred beef cattle^26,33^, which represents a significant proportion of the beef industry^20^. To the best of our knowledge, this is the first study that uses a GWAS to analyze both SC and SM measurements, simultaneously in Canadian crossbred beef cattle. The sample size in the present study (265) might result in a careful interpretation of the obtained results, however many other GWAS have obtained notable results with similar sample sizes, ranging from 41 to 692^15,18,23–27^. For example, Hering et al.^15^ conducted a GWAS on 41 bulls with very poor SM and 279 bulls with excellent SM and identified nine candidate genes all with a strong relationship to sperm function. Buzanskas et al.^26^*.* used SC records on 392 bulls in a GWAS and identified the *STATU2* gene which participates in multiple biological processes, including reproduction, developmental and immune systems. When taken together, the limited studies conducted in crossbred beef cattle, the high heritability of SC and SM, and the evaluation of these traits together results in a study that can bring new considerations for the current stage of the literature regarding male fertility traits in beef cattle.

The ssGBLUP method is based on an infinitesimal model and thus assumes equal variance for all SNP effects, posing an unrealistic situation for traits of economic interest, such as fertility^34^. Therefore, a weighted ssGBLUP (WssGBLUP) approached was used that combines pedigree, phenotype, and genotype data with the integration of different weights for markers in an iterative process to update SNP solutions. For SC, 8 windows, located on BTA9, BTA10, BTA24, and BTA29, explained more than 13.19% of the total genetic variance. For SM, 5 windows explained more than 7.17% of the genetic variance and were located on BTA9, BTA13, BTA20, and BTA24. Of these, BTA9 explained the highest proportion of the variance for SC (4.03%) and SM (2.76%), respectively. Chromosome-wise and genome-wise associations have previously been identified in BTA9 for SC at 420 days in Canchim beef cattle^26^. Moreover, a region on BTA9 in Nellore cattle was found to be associated with testicular hypoplasia, which is defined by a reduced size of both testicles, and consequently reduced scrotal circumference and sperm physiology^35^.

Of the 14 prioritized candidate genes identified for SC (*SASH1, VIP, FBX05, MTRF1L, RGS17, SLC24A5, SEMA6D, MAP3K1, SRPRA, TIRAP, DCPS, ST3GAL4, KIRREL3, CDON*), two, *MAP3K1* and *VIP,* were previously reported as related to male reproduction. Mitogen-activated protein kinase kinase kinase 1 (*MAP3K1*), was mapped within a window that explained 1% of the variance in SM (BTA20: 22.18–23.18 Mb) and was of interest for its anti- and pro-apoptotic functions in germ cells^36^. Guan et al.^37^ found the *MAP3K1* gene was differentially expressed in testis from underfed and well-fed sexually mature sheep, indicating it could be a marker of germ cell apoptosis and therefore change SC and the efficiency of sperm production. The vasoactive intestinal peptide (*VIP;* BTA9: 89.51–90.51 Mb window), another prioritized candidate gene mapped within a window for SC that explained 1.61% of the variance, is known to act directly on the testis, promoting the production of testosterone in mice and rats^38,39^. In a study conducted by Lacombe et al*.*^39^, *VIP* null male mice exhibited a reduction in circulating concentrations of testosterone and follicle stimulating hormone (FSH), inhibiting the morphology of testicular seminiferous tubules. This gene has also been found to play a role in mammalian folliculogenesis, ovarian development, and puberty^5,40,41^.

Five of the prioritized candidate genes for SM have also been previously identified as candidate genes in the regulation of male fertility. These include, superoxide dismutase 2, T-complex protein 1, parkin co-regulated gene, sperm flagella 2 gene, and prolactin receptor (*SOD2, TCP1, PACRG, SPEF2, PRLR*). One of the main causes of sperm chromatin damage is through oxidative stress caused by an imbalance between reactive oxygen species and scavenger systems^42^. Superoxide dismutase isoenzymes, like *SOD2* (BTA9: 95.18–96.18 Mb window), destroy these toxic superoxide radicals that are normally produced within cells^43^. They have been found to be highly expressed in mammalian semen and their activity is positively associated with sperm concentration and motility^44,45^. Another gene found to be associated with spermatogenesis is *TCP1* (BTA9: 95.18–96.18 Mb window), a member of the cytosolic chaperonin-containing *TCP1* complex^46^, which has been identified in the cytosolic fraction^47^ and plasma membrane ^48^ of bovine spermatozoa. The *PACRG* and *SPEF2* genes are essential for the development of normal sperm and male fertility. Previous studies involving *PACRG* (BTA9: 97.25–98.24 Mb window) knockdown in mice have discovered it plays a vital role in maintaining the functional stability of flagella indicating an important relation to sperm motility^49,50^. Moreover, Guo et al*.*^51^ discovered *SPEF2* (BTA20: 38.08–39.08 window) gene expression in the testes and sperm is regulated by alternative splicing and is thus, one of the determining factors of sperm motility. Lastly, *PRLR* (BTA20: 38.08–39.08 window) gene expression was identified in the bovine reproductive tract including, the testis, epididymis, spermatogonia, and differentiating germ cells, leading researchers to believe it may have an effect on spermatogenesis^52^. This gene was also found to play a role in fertilization and survival rates through SNP-SNP interactions^53^.

Five of the prioritized candidate genes for SC and SM have also been identified as playing important roles in the regulation of female fertility. Such genes include, semaphorin 6D, SRP receptor alpha subunit, Kirre like nephrin family adhesion molecule 3, acetyl-CoA Acetyltransferase 2, and proto-oncogene, G protein-coupled receptor (*SEMA6D, SRPRA, KIRREL3, ACAT2, MAS1*). Both *SEMA6D* (BTA10: 22.10–63.21 Mb) and *SRPRA* (BTA29: 29.35–30.35 Mb window) are expressed in the human female genital tract, with *SEMA6D* linked to known gonadal genes^54^ and *SRPRA* upregulated during pregnancy and involved in the transport of secretory and membrane proteins^55^. On the other hand, the *KIRREL3* (BTA29: 29.35–30.35 Mb window), *ACAT2* (BTA9: 95.18–96.18 Mb window), and *MAS1* (BTA9: 95.18–96.18 Mb window) genes have been previously studied in bovine. The *KIRREL3* gene was found to be highly expressed in granulosa cells and may act as a metabolic messenger linking metabolism, body composition and fertility^56^. The *ACAT2* gene was associated with both daughter pregnancy rate and cow conception rate^57^, and *MAS1* gene has been suggested to play a role in the regulation of ovulation^58^. Therefore, despite these genes being identified as prioritized candidate genes regulating male fertility traits, SC and SM, they also play a role in female fertility and regulation.

Through functional analysis, three modules were created with the prioritized candidate genes for SC and SM for the GO:BP, GO:MF, and GO:CC terms related to male fertility and reproduction. Even though nine GO:BP in the module (created via selection of the GO terms related to reproductive processes and the prioritized candidate genes for SC and SM) were related to male fertility and reproduction, due to the few genes contained in the network, only one or two genes were associated with these BPs, thus, none of the terms were significantly enriched. Despite this, it is worthy to highlight the most notable BPs in this network: *regulation of MAPK cascade*, *spermatid differentiation*, and *regulation of hormone secretion*. The MAPK cascade regulates spermatogenesis, sperm maturation, and the acrosome reaction, thereby playing an important role in male reproductive processes^59^. One of the genes involved in the regulation of MAPK cascade is the T-complex protein 1 subunit alpha, *TCP1*, a prioritized candidate gene for SM and is involved in the assembly of actin and tubulin filaments^60^. Actin filaments are present in mammalian germ cells and are involved in a number of changes that occur during spermatogenesis such as, the determination of cell shape, motility, maturation of spermatozoa, and capacitation^61^. In particular, actin proteins change their distribution in the sperm head during maturation and control the balance between globular actin and fibrous actin^61^. Another GO:BP, *spermatid differentiation*, is also an important part of spermatogenesis that involves the differentiation of spermatids into mature spermatozoa in the seminiferous tubules^62^. The prolactin receptor, a SM prioritized candidate gene, was found to be directly linked with spermatid differentiation. Moreover, spermatogenesis and reproductive success is completely dependent on the secretion of various hormones, including FSH, androgen, and testosterone. For example, testosterone levels and SC in bulls are known to be correlated, with peak testosterone levels being lower in bulls with smaller SC^63^. Six of the significant GO:MF were related to male fertility and reproduction, including *acetyltransferase activity*, *zinc ion binding*, *lipase activity*, *endonuclease activity*, *nuclease activity*, and *cation channel activity*. Acetyltransferases, specifically, histone acetyltransferases, act during spermatogenesis through the differentiation of spermatocytes^64^. Zinc is incorporated into spermatozoa where it is bound by seminal fluid Zn-interacting proteins and plays a protective role for sperm chromatin decondensation and sperm motility^65^. Moreover, a number of cation channels, including potassium and chloride channels, are involved in sperm motility, maturation, and the acrosome reaction^66^. Three of the significant GO:CC terms were related to male fertility and reproduction, including, *kinesin complex*, *spindle microtubule* and *cytosol*. A number of kinesin families play key roles in mammalian spermatogenesis, including mitosis, meiosis, acrosome biogenesis, and tail formation^67^. Moreover, kinesins, specifically kinesin-13 s, regulate the spindle microtubule dynamics and control spindle assembly and kinetochore-microtubule attachments^67^. One example of the importance of the cytosol in male fertility is the cytosolic fraction of bovine spermatozoa, which exhibits tyrosine kinase activity associated with sperm capacitation and acrosome reaction^47^. The GO:BP term *spindle microtubule* contained the *TIRAP* and *RGS17* genes which are prioritized candidate genes for SC and *PRLR*, a prioritized candidate gene for SM, indicating that these genes could be important biomarkers of bovine fertility.

The QTL annotation revealed reproduction QTLs account for 7.85% and 8.11% of the QTLs annotated in the windows explaining > 1% of the total genetic variance for SC and SM, respectively. These reproduction QTLs included, *reproductive efficiency*, *age at first calving*, *calving ease*, *daughter pregnancy rate, interval from first to last insemination*, *fertility index*, *conception rate*, and *interval to first estrus after calving*. The largest proportion of QTLs in this study were related to milk production for both SC and SM (31.94% and 54.05%, respectively). A QTL enrichment analysis was conducted as the simple bias of investigation in the QTL database for cattle can result in a larger proportion of records in the database. The enrichment analysis for the windows explaining > 1% of the total genetic variance for SC and SM revealed QTLs annotated for exterior conformation traits, including five different *body depth*, three different *feet and leg conformation*, five different *strength*, and five different *stature* QTLs annotated within the candidate windows for SC, and three different *stature* QTLs and three different *body weight gain* QTLs annotated within the candidate windows for SM (Table 2; Table S5). In a study conducted by Schenkel et al*.*^68^, SC was found to be genetically correlated (*P* < 0.05) to average daily gain, ultrasound backfat thickness, mid-test metabolic weight and hip height in 13,151 bulls (*r*_g_ = 0.24, 0.19, 0.31, 0.16, respectively). Another study revealed a positive correlation for SC and body weight in both pubertal and post-pubertal Holstein bulls (*r*_g_ = 0.76 and 0.45, respectively^69^). Therefore, bulls with larger SC have a larger body size and faster growth^68–70^. This suggests that these regions may be regulating both fertility and conformation traits, however the biological mechanisms associated with this correlation is not well understood^6,71,72^. Other enriched QTLs for SC included *udder depth, udder attachment, teat placement-rear*, and *udder cleft* (Table 2; Table S5). Cows with tightly attached udders and proper teats tend to remain in a herd longer, are easier to nurse, and less susceptible to mastitis, therefore udder traits may be used in conjunction with body conformation traits for the indirect selection of longevity in beef cattle^73,74^. The QTL enrichment analysis for SM also revealed QTLs related to fertility on BTA9, one for *daughter pregnancy rate* and two different QTLs for interval to *first estrus after calving* (Table 2; Table S5). This is consistent with other studies that reveal a genetic correlation between male and female fertility traits. For example, Johnston et al*.* ^75^ found SM at 18 months of age to be highly correlated with female reproduction traits in Brahman and Tropical Composite cows, such as conception (0.53 and 0.72, respectively) and pregnancy rate (0.58 and 0.95, respectively). Gargantini et al*.*^76^ identified a genetic correlation between yearling SC and age at puberty and pregnancy rate in heifers was − 0.57 and 0.35, respectively. Thus, BTA9 may be a candidate region for bovine fertility. A study conducted by McClure et al*.*^77^ identified 41 SC QTLs in Angus cattle, of which three QTLs were identified on BTA9. The overlap between the candidate regions identified in the present study and previous studies reinforces the association of these genomic regions with the regulation of genes and biological processes responsible for male fertility traits, including *VIP* and *SOD2* genes*,* mapped on BTA9, and the *regulation of MAPK cascade* and *spermatid differentiation.*

To conclude, functional analysis for the prioritized candidate genes identified in this study revealed significant GO terms associated with biological processes and molecular functions related to male fertility and reproduction. For scrotal circumference, both *MAP3K1* and *VIP* identified genes control testis function and could be used as potential biomarkers of spermatogenesis and apoptosis. For sperm motility, the *SOD2, TCP1, PACRG, SPEF2,* and *PRLR* genes were related to sperm concentration, development, and motility. These results help to better understand the genetic bases of scrotal circumference and sperm motility specifically in crossbred beef bulls and revealed positional candidate genes with additional functional evidence that might ultimately improve bull genomic prediction for these traits. Moreover, these candidate regions, specifically those mapped on BTA9, have known genetic correlations to other economically important traits including, conformation, female fertility, and udder structure. However, future research on these candidate genes and their impact on bull fertility is warranted.

## Materials and methods

### Population structure and phenotypic data

Data used in this study are from animals cared for under protocols approved by the University of Guelph Animal Care Committee, which follows guidelines of the Canadian Council on Animal Care (1993).

The population for this study came from the Ontario Beef Research Centre; the University of Guelph research farm located in Elora, ON, Canada and consisted of 265 crossbred bulls. At the time of collection, bulls had an average age of 384 days and average weight of 555 kg. The predominant breeds (and corresponding average composition of that breed in the test group, see Table S1) of these crossbred bulls were Angus (AN: 52%), Simmental (SM: 24%), Piedmontese (PI: 6.6%), Gelbvieh (GV: 6.3%), Charolais (CH: 3.8%), and Limousine (LM: 1.1%).

The fertility traits used in this study were SC and SM. Scrotal circumference was measured by palpating the testes into the lower half of the scrotum and measuring the greatest circumference using a looped tape as described by Awada et al*.*^19^. Semen ejaculates were collected between 12 and 15 months of age immediately after the SC measurement using electroejaculation (Pulsator IV- Auto Adjust Electro-Ejaculator: Lane Manufacturing, Inc, Denver, CO). Sperm motility was visually estimated immediately and ejaculates were then extended, cooled, placed in liquid nitrogen and thawed as previously described by Pursel & Johnson^78^. Sperm motility was assessed as described by Awada et al*.*^79^. Briefly, sperm were evaluated on the CASA (Integrated Visual Optical System (IVOS) CASA System, Hamilton Thorne, Inc., Beverly, MA) sampled from 1 × 10^7^ spermatozoa/ml in BTS^78^ and a four-chamber standard count analysis slide (Leja products B.V. Luzernestraat 10, 2153 GN Nieuw-Vennep, The Netherlands), at 37 °C.

### Genotyping and quality control

Genotyping was completed from 265 animals using the Affymetrix Genechip Bovine Genome High Density Array, which included 648,874 SNPs. Marker coordinates were converted to the new bovine genome assembly ARS-UCD 1.2. Quality control was performed using Plink^80^ and the following criteria were used for the exclusion of SNPs: non-autosomal regions; minor allele frequency < 0.05; and a call rate < 95%.

### Genome-wide association study

The programs of BLUPF90 family were used for the weighted single-step genomic BLUP (WssGBLUP) analysis^81^. The GWAS results were reported as the proportion of the variance explained by non-overlapping genomic windows of 1 Mb^82^.

A WssGBLUP method was used to estimate SNP effects. The observed phenotypes of SC and SM were used as dependent variables in a single-trait animal model:

$$y = Xb + Z_{a} a + e$$

where y represented a vector of observed phenotypes for animals (SC or SM); **X** is the incidence matrix of fixed effects; *b* is the vector of fixed effects, **Z**_***a***_ is the incidence matrix of additive genetic effects; *a* is the random vector of additive genetic effects; and *e* is the vector of residual effects. Fixed effects for SC included 25 levels of herd-year-season (HYS), body weight, age as a third-degree polynomial, and breed composition for the most prevalent breeds (AN, SM, PI, GV, CH, and LM). Fixed effects for SM included 25 levels of HYS, age, and breed composition for the most prevalent breeds.

The solutions of the SNP effects (*û*) were obtained using the AIREMLF90^81^ algorithm with two iterations, as proposed by Wang et al*.*^34^.

For each iteration of the algorithm D_(*n*)_ = I and G_(*n*)_ = ZD_(*n*)_*Z’λ*, where D is a diagonal matrix of weights for SNP variances, G is the weighted genomic relationship matrix, Z is a matrix relating genotypes of each locus, *λ* is a variance ratio, and *n* is the iteration number; the breeding values (â_*v*_) were calculated using single-step genomic best linear unbiased predictor (ssGBLUP); SNP effects were calculated via û_(*n*)_ = λD_(*n*)_Z′G_(*n*)_^−1^â_*v*_; SNP weights were calculated via d_i(*n*+1)_ = û^2^_i(*n*)_2p_*i*_(1 − p_*i*_), where i is the ith SNP; the weights were normalized via D_(*n*+1)_${ } = \frac{{tr(D_{\left( 0 \right)} )}}{{tr(D_{{\left( {n + 1} \right)}} )}}$_D(*n*+1)_ so the additive genetic variance remains constant; and the Genomic matrix was recalculated via G_(*n*+1)_ = ZD_( *n*+1)_*Z’λ* to obtain the SNP effects. These iterations were used to calculate the proportion of variance explained by non-overlapping windows.

The proportion of variance explained by non-overlapping windows were estimated using the PostGSf90 algorithm by summing the variance of SNPs within 1 Mb^82^. Windows that explained greater than 1% of the genetic variance for SC and SM were selected for QTL and gene annotation^83^, conducted using R (Version 4.0.0.; R Core Team, 2020) and the R package: Genomic Annotation in Livestock for positional candidate LOci (GALLO—https://github.com/pablobio/GALLO). The .gtf annotation file corresponding to the bovine gene annotation from ARS-UCD1.2 assembly and the .gff file with the QTL information from Animal QTL Database^84^, using the same reference genome (ARS-UCD1.2) to map the QTLs, were used for gene and QTL annotation, respectively. A QTL enrichment analysis was also conducted using the GALLO R package for all the QTL information annotated within the candidate windows using a chromosome-based enrichment analysis. Briefly, a bootstrap approach was used to compare the observed number of QTL for each trait in each chromosome annotated using GALLO with the expected number for each trait estimated using 1000 iteration rounds of random sampling from the whole Cattle QTLdb. Using this approach, a *p *value for the QTL enrichment status of each annotated trait within the candidate windows was calculated, Additionally, the *p *values were corrected for multiple testing using FDR (5%).

### Gene prioritization analysis

Functional candidate gene prioritization was conducted using the ToppGene Suite^85^. A trained list of genes associated with keywords outlined by Fonseca et al*.* ^21^ using the GUILDify^86^ software and a species-specific (*Homo sapiens*) interaction network, including, “scrotal circumference,” “scrotal,” “testicular,” “testis,” “testes,” “sperm,” “semen,” “spermatozoa,” “spermatogenesis,” and “fertility,” made up the “trained list” of genes. From this analysis, the top 100 genes ranked using an algorithm based on network topology on GUILDify were used to create the “trained” gene list. This “trained” gene list was used in conjunction with the “test” gene list containing the genes within windows explaining greater than 1% of the genetic variance for SC and SM. An annotation-based prioritization analysis through a multivariate approach was conducted using ToppGene. Gene Ontology terms for molecular function (MF), biological process (BP), and cellular component (CC); human and mouse phenotypes; metabolic pathways; Pubmed publications; and diseases were utilized to retrieve functional information for the trained list and for the list of positional candidate genes (those genes annotated within windows explaining more than 1% of the genetic variance for the trait). Overall *p *values were obtained using a combination of the *p *values obtained from the intermediate values from the above functional information using a random sampling of 5000 genes from the whole genome for each annotation information. Significant prioritized genes were selected based on a FDR 5% multiple correction, meaning these genes have a functional profile that is significantly similar to the functional profile of the “trained” gene list. As the “trained” gene list is known to be associated with fertility, by the guilty by association principle, it is probable that the prioritized genes will also be associated with fertility.

### Functional analysis

A protein–protein interaction network analysis was performed in order to identify interactions between the positional candidate genes and other genes in the genome that are relevant to fertility. Therefore, the prioritized candidate genes for both SC and SM were inputted together into NetworkAnalyst 3.0 (^87^ https://www.networkanalyst.ca) and the STRING interactome protein–protein interaction database was used with a confidence score cutoff of 900. A second-order interaction network was generated containing 1442 nodes (genes) with 4888 edges. Then, gene ontology (GO) enrichment analyses, including the three main categories biological process (BP), molecular functions (MF), and cellular components (CC), was performed using the genes comprising the network^88^. Functional evidence of the relationship between the significant GO terms (FDR-corrected *p* value ≤ 0.05) and the target phenotypes (SC and SM) was identified. The GO terms related to reproductive processes were selected to extract the first module from this network composed only by the nodes (genes) associated with the selected GO:BP, GO:MF and GO:CC. Next, a second module was extracted, from the previous sub-network. This second module was composed only by the positional prioritized candidate genes for SC and SM and its direct connected nodes. The GO:BP, GO:MF and GO:CC enriched for this second module were analyzed for its association with reproductive processes.

## Supplementary information

Supplementary Table S1.

Supplementary Table S2.

Supplementary Table S3.

Supplementary Table S4.

Supplementary Table S5.

Supplementary Information 6.

## Abbreviations

ssGBLUP: Single-step genomic BLUP
WssGBLUP: Weighted single-step genomic BLUP
SC: Scrotal circumference
SM: Sperm motility
FDR: False discovery rate
AN: Angus
SM: Simmental
PI: Piedmontese
GV: Gelbvieh
CH: Charolais
LM: Limousine
HYS: Herd-year-season
GALLO: Genomic Annotation in Livestock for positional candidate LOci
MF: Molecular function
BP: Biological process
CC: Cellular component
MAP3K1: Mitogen-activated protein kinase kinase kinase 1
VIP: Vasoactive intestinal peptide
FSH: Follicle stimulating hormone
SOD2: Superoxide dismutase 2
TCP1: T-complex protein 1
PACRG: Parkin co-regulated gene
SPEF2: Sperm flagella 2 gene
PRLR: Prolactin receptor
SEMA6D: Semaphorin 6D
SRPRA: SRP receptor alpha subunit
KIRREL3: Kirre like nephrin family adhesion molecule 3
ACAT2: Acetyl-CoA Acetyltransferase 2
MAS1: Proto-oncogene, G protein-coupled receptor
TCP1: T-complex protein 1 subunit alpha
