# Supplementary material for: Genome-wide association study to identify genomic regions and positional candidate genes associated with male fertility in beef cattle
Source: Sci Rep. 2020 Nov 18;10:20102. doi: 10.1038/s41598-020-75758-3 (PMC7676258; doi:10.1038/s41598-020-75758-3)
Supplement: Supplementary file 6 — Supplementary Information 6. [file 41598_2020_75758_MOESM6_ESM.docx]

Running head: GWAS for male fertility traits in beef cattle

**Genome-wide association study to identify genomic regions and positional candidate genes associated with male fertility in beef cattle**

**H. Sweett^*^, P.A.S Fonseca^*^, A. Suárez-Vega^*^, A. Livernois^*,†^, F. Miglior^*^, A. Cánovas^*,1^**

*Centre for Genetic Improvement of Livestock, Department of Animal Biosciences, University of Guelph, Guelph, Ontario, N1G 2W1, Canada

†Department of Pathobiology, Ontario Veterinary College, University of Guelph, Guelph, Ontario, N1G 2W1,Canada

^1^Corresponding author: [acanovas@uoguelph.ca](mailto:acanovas@uoguelph.ca)
